# Quantitative utilization of prior biological knowledge in the Bayesian network modeling of gene expression data

**DOI:** 10.1186/1471-2105-12-359

**Published:** 2011-08-31

**Authors:** Shouguo Gao, Xujing Wang

**Affiliations:** 1 Department of Physics, University of Alabama at Birmingham, 1300 University Blvd, Birmingham, AL 35294, USA; 2 The Comprehensive Diabetes Center, University of Alabama at Birmingham, 1825 University Blvd, Birmingham, AL 35294, USA

## Abstract

**Background:**

Bayesian Network (BN) is a powerful approach to reconstructing genetic regulatory networks from gene expression data. However, expression data by itself suffers from high noise and lack of power. Incorporating prior biological knowledge can improve the performance. As each type of prior knowledge on its own may be incomplete or limited by quality issues, integrating multiple sources of prior knowledge to utilize their consensus is desirable.

**Results:**

We introduce a new method to incorporate the quantitative information from multiple sources of prior knowledge. It first uses the Naïve Bayesian classifier to assess the likelihood of functional linkage between gene pairs based on prior knowledge. In this study we included cocitation in PubMed and schematic similarity in Gene Ontology annotation. A candidate network edge reservoir is then created in which the copy number of each edge is proportional to the estimated likelihood of linkage between the two corresponding genes. In network simulation the Markov Chain Monte Carlo sampling algorithm is adopted, and samples from this reservoir at each iteration to generate new candidate networks. We evaluated the new algorithm using both simulated and real gene expression data including that from a yeast cell cycle and a mouse pancreas development/growth study. Incorporating prior knowledge led to a ~2 fold increase in the number of known transcription regulations recovered, without significant change in false positive rate. In contrast, without the prior knowledge BN modeling is not always better than a random selection, demonstrating the necessity in network modeling to supplement the gene expression data with additional information.

**Conclusion:**

our new development provides a statistical means to utilize the quantitative information in prior biological knowledge in the BN modeling of gene expression data, which significantly improves the performance.

## Background

Reverse engineering of genetic networks will greatly facilitate the dissection of cellular functions at the molecular level [1-3]. The time course gene expression study offers an ideal data source for transcription regulatory network modeling. However, in a typical microarray experiment usually up to tens of thousands of genes are measured in only several dozens or less samples, data from such experiments alone is significantly underpowered, leading to high rate of false positive predictions [4]. Network reconstruction from microarray data is further limited by low data quality, noise and measurement errors [5].

Incorporating other types of data and existing knowledge of gene relationships into the network modeling process is a practical approach to overcome some of these problems. It has been proven that data integration and useful bias with relevant knowledge can improve the network prediction accuracy from gene expression data [6,7]. Among the various approaches of network modeling, Bayesian Networks (BN) have shown great promise and are receiving increasing attention [8]. BN is a graphic probabilistic model that describes multiple interacting quantities by a directed acyclic graph (DAG). The nodes in the network represent random variables (expression levels), and edges represent conditional dependencies between nodes [9]. Learning a BN structure is to find a DAG that best matches the dataset, namely maximizing the posterior probability of DAG given data D: *P *(DAG|D). The sound probabilistic schematics allow BN to deal with the inherent stochasticity in gene expressions and the noise brought in by the microarray technology. Furthermore, BN is capable of integrating prior knowledge into the system in a natural way [9,10].

A number of studies demonstrated that adding prior knowledge to BN improved the performance [4,11-14]. Many sources of data and information are useful to supplement the gene expression data, and they can be incorporated at different steps of BN simulation, from prior structure definition to structure simulation and evaluation.

Known protein-DNA interaction or other clues of the relationships between transcription factors and their target genes are useful to transcription regulatory network inference. Hartemink *et al*. included data from the chromatin immunoprecipitation (ChIP) assay [15], and Tamada *et al *incorporated promoter sequence motif information [16], to define the prior probability of network structures. Information of other types of gene pair relationship has also been explored. Steele *et al*. developed a gene-pair association score from the correlation of their concept profiles derived from literature, and utilized that to define the prior structure probabilities [12]. Larsen *et al *defined a Likelihood of Interaction (LOI) score, which measures the statistical significance of two genes interacting with each other according to their shared Gene Ontology (GO) information. They then restricted the candidate network edges (interactions) to those with significant *p *-values of LOI during the BN structure learning iterations [17,18]. By doing so, the quantitative information of the likelihood is not fully utilized in the network modeling. Djebbari and Quackenbush utilized literature, high-throughput protein-protein interaction (PPI) data, or the combination of both to define the seed (initial) network structure. They observed an improved ability of the BN analysis to learn gene interaction networks from the expression data [19].

Imoto *et al *formulated an novel approach to incorporate prior biological knowledge within the BN framework by adopting the energy concepts from statistical physics [20,21], which was later further extended by Husmeier and Werhli [22,23]. In this approach an energy function was first defined to measure the agreement between a candidate network and the prior biological knowledge, and prior distribution of network structure is hence calculated using the Gibbs distribution in a canonical ensemble. Using this approach, the two groups examined several types of prior knowledge, including PPI, protein-DNA interaction, binding site information, literature, and Kyoto Encyclopedia of Genes and Genomes (KEGG) pathways [22-24]. The algorithms were validated using yeast gene expression data [20,21], and synthetic data [22].

Existing studies often utilize prior knowledge to construct the prior distribution of network, or initial network structure. It has been demonstrated that the sampling method during simulation also affects the performance of BN structure learning [25]. Though prior knowledge has been utilized to bias the sampling step, it is normally done through restricting the search space to sub regions, for instance, only simulate candidate structures whose significance is above a certain threshold according to prior knowledge [17,18].

In searching for the network structure (DAG) that maximize *P *(DAG|D), the Markov Chain Monte Carlo (MCMC) approach is regarded better than greedy searching algorithms, especially for the microarray data with small sample size where there is often no single structure that is prominently better than others [9]. In this study we propose a new approach to incorporate prior knowledge in a quantitative way to bias the MCMC simulation of candidate structure. It utilizes information of functional linkage between gene pairs, assuming that functionally linked genes are likely to interact with each other. It is known that interacting proteins or genes often share similar function, and participate in the same biological pathways and processes [26]. Interaction has been utilized to infer functional linkage and annotate gene functions [27]. Increasing evidence suggests that the reverse is also frequently true [28]. In our algorithm a probability score is first calculated that measures how likely two genes are functionally linked based on prior knowledge; A candidate edge reservoir is then constructed where the number of copies of each edge is proportional to this probability score; The reservoir is in turn used for sampling candidate network structure during the MCMC simulation. This way the quantitative information of the potential gene pair link predicted by prior knowledge is retained.

We will consider two type of prior knowledge: co-citation in PubMed literature and similarity in ontological annotation according GO http://www.geneontology.org/. We will demonstrate they both contain information of functional linkage. The performance of the new algorithm is evaluated using a synthetic data set as well as data from two real microarray experiments: the yeast cell cycle study, and the mouse pancreas development/growth study. We will demonstrate that including the prior knowledge significantly improves the performance of BN modeling of gene expression data.

## Results

### Algorithm

**Figure 1:**
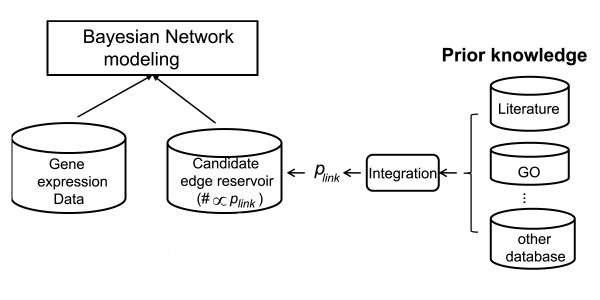
**The framework of our BN modeling to incorporate the quantitative information in prior knowledge**.

BN is a graphical model to capture complex relationships among a set of random variables {*X*_1_, *X*_2_,...,*X_n_*} encoding the Markov assumption, each node representing a variable. In the context of gene network modeling, each node represents a gene, while gene interactions are represented by directed edges between nodes. Each variable *X_i _*in the DAG is conditionally independent of its non-descendants given its set of parents. Mathematically the joint distribution of the DAG can be decomposed into a product form as:

(1) $$P\left(\text{DAG}\right)=P\left(X_{1},X_{2},\ldots ,X_{n}\right)=\overset{n}{\underset{i=1}{\prod }}P\left(X_{i}∕\Pi_{i}\right)$$

where Π*_i _*denotes the parent set of the variable *X_i_*. This is referred as the chain rule for BNs [9,10]. Learning a BN structure is to find a DAG that best matches the dataset, namely maximizing the posterior probability of DAG given data *P *(DAG|D). Here we adopt the sampling-based approach to Bayesian inference, and sample network structures from a candidate edge reservoir with the MCMC network learning method. In the reservoir the edge representation is proportional to the likelihood of the two genes being functionally linked according to prior knowledge. This way, the edges between the strongly-related gene pairs have higher chance to be proposed as part of the candidate network. The overall design is given in Figure 1. The major steps included:

1. Determine the probability of functional link *p*_link _between each gene pairs

1.1 Calculate GO schematic similarity

1.2 Calculate p value of PubMed co-citation.

1.3 Integrate GO and PubMed information using the Naïve BN to determine *p*_link_.

2. Construct candidate network edge reservoir in which copy number of each edge is proportional to the *p*_link _of the corresponding gene pair.

3. Learn network structure using the MCMC algorithm through sampling the candidate network edge reservoir.

At each step of the iteration, the proposed network is retained with an acceptance probability that is determined by the relative posterior of the proposed versus current network, penalized by the network complexity [29,30]. In calculating the posterior we use the BDe (Bayesian Dirichlet equivalence) scoring metric [10,31]. The prior distribution is assumed to be uniform.

To evaluate the performance of our BN algorithm, and the benefit of adding prior knowledge, we compare it to two alternative approaches: (1) Plain BN. In each iteration, a new network is proposed by randomly changing one edge in the current network. (2) The method developed by Husmeier and Werhli [22,23].

### GO schematic similarity and significance of PubMed co-citation

GO annotation and gene citation database (PubMed) were downloaded from ftp://ftp.ncbi.nlm.nih.gov/gene/DATA. Schematic similarity in GO taxonomy was first calculated for each gene pair using the approach proposed by Cao *et al *[32], which calculates the shared information content of the GO terms. The value of this measure ranges between [0 1], with 0 being no similarity, and 1 being maximum similarity. The GO similarity between each gene pair is defined to be the maximum schematic similarity of all the GO terms they share.

For a given pair of genes, the total number of PubMed abstracts in which each gene appears (*n *and *m*, respectively), and in which both appear (*k*) were determined. The probability of co-citation frequency observed by random chance is calculated by

(2) $$p_{\text{PubMed}}\left(\#\;\text{of}\;\text{co - citation}\geq k|n,m,N\right)=1-\overset{k-1}{\underset{i=0}{\sum }}p\left(i|n,m,N\right)$$

where $p\left(i|n,m,N\right)=\frac{n!\left(N-n\right)!m!\left(N-m\right)!}{\left(n-i\right)!i!\left(m-i\right)!\left(N-n-m+i\right)!N!}$, and N is the total number of abstracts in PubMed [1,2].

### Construction of the candidate network edge reservoir

We used the Naïve Bayesian network to integrate the GO and co-citation information, and a simple Bayesian naïve classifier to predict the functional linkage probability *p_link _*for all gene pairs. Note that the prior knowledge of functional linkage is undirected, *i.e. p_link _*(*i*, *j*) *= p_link _*(*j*, *i*). An edge sampling reservoir was constructed, in which the number of replicates for the edge between gene *i *and *j N *(Edge*_i_*_,_*_j_*) is in proportion to their *p*_link _:

(3) $$N\left(\text{Edg}e_{i,j}\right)=\text{Ceil}\left(10\times p_{\text{link}}\left(i,j\right)\right)$$

where Ceil(*x*) is the smallest integer no less than *x*. In this definition, any gene pair will be represented at least once and at most 10 times. The edges of gene pairs with higher *p*_link _will appear more frequently in the edge reservoir, and hence enjoy a higher chance to be selected during the network structure learning.

### Implementation

Our BN simulation algorithm is implemented in Matlab utilizing Kevin Murphy's BNT package [33]bnt.googlecode.com, and is summarized in Table 1. Note that steps 1 and 3.1 contain unique features that separate our approach from others. The source code is available upon request. The networks were visualized with Cytoscape [34].

**Table 1:** Implemention of the new BN structure learning algorithm

**Input:**
n: number of nodes in the network.
D: discretized expression data matrix.
BurnIn: number of steps to take before drawing sample networks for evaluation. Default value: 50 times the size of the sampling reservoir.
n_iteration: number of iterations. Default value: 80 times the size of the sampling reservoir.
Δ_samples: interval of sample networks being collected from the chain after burn-in. Default
value: 1000.
maxFanIn: maximum number of parents of a node.

**Output:**
A set of DAGs after reaching the max iteration step.
An average DAG in the form of a matrix.

**Steps**
1. Create a sampling edge reservoir based on *p_link_*.
2. Set all elements of the adjacency matrix for the initial DAG to 0.
3. for loop_index = 1: n_iteration do
(1) randomly select a element edge(i,j) from the edge sampling reservoir, corresponding to gene pair (i,j).
(2) if edge(i,j) exists in the current DAG, delete the edge; else if edge(j,i) exists in the current DAG, reverse edge(j,i) to edge(j,i); else add edge(i,j). We name these operations as "delete", "reverse" and "add", respectively.
(3) check whether the newly proposed DAG remains acyclic and satisfy the maxFanIn rules to nodes (i,j). If not, keep the current DAG and give up proposed DAG, go to (1).
(4) calculate log value of the marginal likelihood (LL)* of the expression data D of node j and its parents given the current DAG (LL_old) or the proposed DAG (LL_new) and define bf1 = exp(LL_new - LL_old).
(5) if the operation is "delete" or "add", bf2 = 1; if the operation is "reverse", calculate bf2 for node i in same way as for node j in (4).
(6) calculate the prior probability* of current DAG (prior_old) and propose DAG (prior_new); calculate the Metropolis-Hastings ratio (*R*_HM_) of the two DAGs; generate a random number u between 0 to 1, if bf1*bf2*prior_new/prior_old<u*R_HM_, keep the current DAG and give up proposed DAG, go to (1).
(7) when loop_index>BurnIn and (loop_index-BurnIn) is exactly divisible by **Δ**_samples, record the proposed DAG and its posterior probability.
4. End of loop, calculate the average DAG in the form of a matrix, where the elements are given by the averaged edges of all recorded DAGs weighted by their posterior probabilities.

*Details of the definition of marginal likelihood, and how to calculate LL, prior probability of DAG, can be found in [10,31].

### Validation

#### Utility of GO similarity and PubMed co-citation in discovering functional linkage between gene pairs

Lee *et al *developed an approach to evaluate if gene-pair functional relationships can be predicted by a certain type of high-throughput genomic data (gene expression, PPI, ChIP-chip, etc) [35,36]. Assuming that *p*(*L*|*D*) and (~ *L*|*D*) denote the probabilities of gene pairs to share or not share functional annotation given that they are linked by data *D *(for instance, co-expressed, sharing PPI, protein of one gene binds to the promoter of the other, etc), and *p*(*L*) and p(~ *L*) represent the prior probabilities of sharing and not sharing functional annotation, they proposed a log likelihood score [35,36]:

(4) $$LLS=\text{ln}\left(\frac{P\left(L|D\right)∕P\left(\sim L∕D\right)}{P\left(L\right)∕P\left(\sim L\right)}\right)$$

to describe the utility of data *D *in functional linkage inference. An LLS close to 0 suggest that the data is not more informative than random pairing, whilst higher positive values of LLS indicates that data *D *contains more information of functional linkage.

We adopted equation (4) to evaluate whether GO schematic similarity and PubMed co-citation were useful in identifying functional linkage. The KEGG http://www.genome.ad.jp/KEGG and Munich Information Center for Protein Sequences (MIPS, mips.gsf.de/) database [1,2] were used to construct the benchmarks of functional linkage. These databases were chosen for their high quality [37]. In this study we utilized yeast and mouse gene expression data to validate our algorithm. For each species, the positive control set consists of randomly sampled 5% (43,761 for yeast, and 35,424 for mouse) of all gene pairs that are in the same KEGG pathways [38]. The choice of 5% rather than all is to lower the computational complexity. The negative control set was constructed with gene pairs that encode proteins localized in different cellular compartments, with the underlying assumption that they are functionally unrelated and do not interact with each other. Four categories in the MIPS annotation [39] were utilized: 70.03 cytoplasm, 70.10 nucleus, 70.16 mitochondrion, and 70.27 extracellular/secretion proteins.

Again we only kept 5% of all possible gene pairs, totaling 112,693 for yeast and 531,089 for mouse, respectively. The same benchmark sets were also utilized to train the Naïve Bayesian classifier when calculating *p*_link_.

The LLS of co-citation in discovering functional linkage is then determined by:

(5) $$LLS_{\text{PubMed}}=\text{ln}\left(\frac{P\left(L|p_{\text{pubMed}}\right)∕P\left(\sim L∕p_{\text{pubMed}}\right)}{P\left(L\right)∕P\left(\sim L\right)}\right)$$

The LLS of GO schematic similarity was performed in the similar fashion. The LLS value for gene pair sets in different ranges of GO similarity and co-citation p-value were given in Table 2. Gene pairs sets with higher GO similarity or PubMed co-citation significance, have more positive LLS values, and vice versa. Note that gene pairs with negative LLS means they are less likely to be functionally linked than random pairs, which is expected if they share low GO similarity or co-citation. The results suggest that PubMed Co-citation and GO similarity are efficient at discriminating functionally linked gene pairs from not linked ones.

**Table 2:** GO and PubMed citation contain information of functional linkage

interval	GO similarity LLS, yeast	LLS, mouse	interval	-log_10_(p_PubMed_)LLS, yeast	LLS, mouse
[1, 1]	1.51	1.62	(4 ∞)	0.25	0.37
[0.2, 1)	-0.71	-0.99	(3 4]	0.13	0.14
[0, 0.2)	-1.61	-2.2	(1 3]	0.07	0.19
			[0 1]	-3.4	-3.6

Log Likelihood Scores of functional linkage in yeast and mouse, for gene pair in different value interval of GO similarity and PubMed co-citation significance. Gene pairs with higher GO similarity or significance of co-citation are more likely to be functionally linked.

We found that there is a marginal dependence between the GO similarity and PubMed co-citation (Fisher's Z test, p~0.1). Theoretically naïve Bayesian classifier is optimal when the attributes are independent given class. However, empirical studies have shown that the classifier still performs well in many domains when there is moderate attribute dependences [40]. The weak dependence between them indicates that the naïve Bayesian Network is an appropriate choice to integrate their information [41]. Interestingly, the GO and MIPS categories, which are both functional annotations, also only depend weakly on each other. This may be because the present annotations are far from being perfect and complete [42].

### Utility of functional linkage information to interaction network modeling

**Figure 2:**
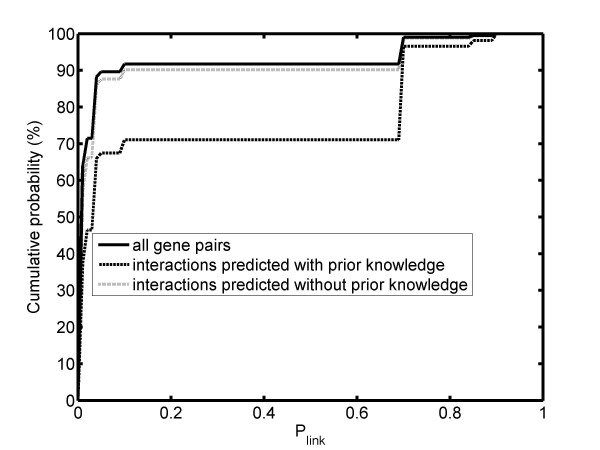
**Distribution of functional linkage probability for all possible gene pairs, and for predicted interactions with and without prior knowledge**.

The distribution of *p_link _*for yeast gene pairs is given in Figure 2. Note that only a small proportion of gene pairs share high values of *p_link_*, for about 92% of the gene pairs this value is less than 0.2. This indicates that most gene pairs share no functional linkage, consistent with the fact that gene networks are usually sparse. The candidate edge reservoir is constructed according to equation 3, and the MCMC samples this distribution to propose new candidate network structure at each iteration. In Figure 2 we have also included the distribution for gene pairs predicted to be interacting to each other, with and without the prior knowledge. Among all possible gene pairs, only ~8% with *p_link _*0.6. In contrast, this proportion increases to 28% among the predicted interactions. It indicates that the prior knowledge did affect the outcome of the BN learning. The results from the other data sets are similar.

The assumption of incorporating prior knowledge of functional linkage is that they can help network modeling. Existing data from yeast revealed that genes sharing the same GO attribute interact genetically more often than expected by chance (p < 0.05) [43,44]. In a very conservative estimate, over ~12% of the genetic interactions are comprised of genes with identical GO annotation (a 12 fold enhancement over what expected by chance, p < 10^-12^); and over 27% are between genes with similar or identical GO annotations (an 8 fold enhancement, *p *< 10^-10^).

**Figure 3:**
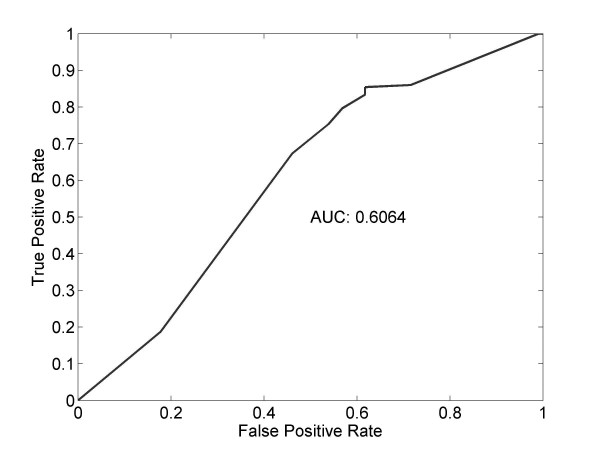
**ROC curve indicating that functional linkage contains information for interaction**. Plotted is the performance of *p_link _*as a classifier to identify yeast TF-target pairs defined by ChIP-chip.

We examined whether *p_link _*can potentially discriminate interacting gene pairs from non-interacting ones, using the receiver operating characteristic (ROC) curve. ROC is a graphical plot of the sensitivity versus (1-specificity), namely the fraction of true positives versus the fraction of false positives, as the discrimination threshold of a classifier is varied. The area under curve (AUC) reflects the performance. The ROC of a random classifier would be a 45° line with AUC = 0.5. Figure 3 presents the ROC plot for the nine yeast cell cycle regulating transcription factors (TF): Fkh1, Fkh2, Ndd1, Mcm1, Ace2, Swi5, Mbp1, Swi4, and Swi6, and their targets identified using the ChIP-chip technology [45]. The AUC of 0.6064 indicating that is positively correlated with interaction and therefore useful in interaction inference.

### Convergence of simulation

**Figure 4:**
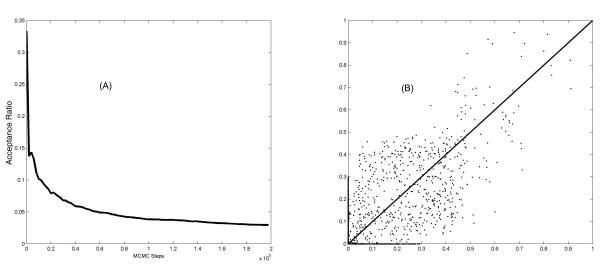
**Convergence of simulation**. (A) Acceptance ratio versus the number of MCMC steps (B) scatter plot of the marginal posterior probabilities of the edges, obtained from two separate MCMC simulations of the yeast cell cycle data.

In Figure 4A we plot the acceptance ratio versus number of MCMC steps in the yeast cell cycle dataset. Obviously in the later steps the probability to accept the new proposed DAG is small and flattens. The results from the other datasets are similar. In addition, the MCMC simulation was repeated 20 times with independent initializations, and consistency in the marginal posterior probabilities was examined. We found that they correlated well between different runs: 0.83 ± 0.11 for the simulated dataset, 0.68 ± 0.10 for the yeast data set, and 0.51 ± 0.26 for the mouse pancreas dataset. Figure 4B presents the scatter plot of the edge posterior probability from two typical runs that simulate the yeast dataset.

### Validation using simulated data

In our network inference, the MCMC learning simulation is repeated 20 times with independent initializations and an interaction will be considered in the final network if it is observed more than 15 times. Our new BN algorithm was first tested in a simulated time course (50 time points) gene expression dataset of an artificial network generated using SynTReN [46]. This network contains 76 genes, of which 24 act as regulators with a total of 124 regulatory relationships (*i.e*. 124 edges). The results are summarized in Table 3, 2n^d ^column. It demonstrates that incorporating the functional linkage as prior knowledge allows the identification of a significantly higher number, 21 versus 14, of the true gene-gene relationships compared with the plain BN modeling of gene expression data only. A random network of the same number of edges was also created for the 76 genes [47]. The improvement of BN with prior knowledge over random is significant (p < 0.01, Table 3), while without prior knowledge it is not (p~0.2, Table 3).

**Table 3:** The improvement in network modeling with the addition of prior knowledge

Data set	Simulated data	Yeast cell cycle study, benchmark from BIND	Yeast cell cycle study, benchmark from ChIP-chip	Mouse pancreas study
Number of genes	76	107	107	36
Number of established regulations	124	114	190	24
Number of possible regulations	76*75 = 5700	107*106/2 = 5671*	9*106 = 954	36*35 = 1260
Number of known regulations recovered with (without) prior knowledge	21 (14)	26 (13)	23 (11)	12 (6)
Total number of regulations predicted, with (without) prior knowledge	503 (440)	436 (387)	58 (33)	322 (297)
Improvement over plain BN	χ ^2 ^= 0.36, p~0.54	χ ^2 ^= 2.28, p < 0.13	χ ^2 ^= 0.04, p~0.84	χ ^2 ^= 0.98, p~0.32
Improvement: over random selection	χ ^2 ^= 7.32, p < 0.01	χ ^2 ^= 24.5, p < 0.001	χ ^2^= 6.71, p < 0.01	χ ^2 ^= 2.87, p < 0.09
Plain BN over random selection	χ ^2 ^= 1.58, p~0.2	χ ^2 ^= 2.42, p~0.11	χ ^2 ^= 1.6, p~0.2	χ ^2 ^= 0.01, p~0.8

* We ignored edge direction with comparing to BIND since it contains both directed and undirected interactions.

### Validation using the yeast cell cycle data

Next the new algorithm was applied to one of the Stanford yeast cell cycle data http://genome-www.stanford.edu/cellcycle/, where the cells from a cdc15 temperature sensitive mutant were studied [48]. To evaluate the performance, we compared the predicted interactions from our algorithm to the annotated interactions in BIND http://bind.ca[49], and the transcription regulation predicted by the ChIP-chip data [45]. Tables 4, 5 list the benchmark interactions for the 107 yeast cell cycle genes that were recovered by the BN modeling. The statistical results are summarized in Table 3, columns 3-4.

**Table 4:** Predicted yeast gene regulatory relationships that are annotated in BIND

**BN with prior knowledge**			
**HTA1**→**HHT1**	**FUS1**→**FAR1**	**FKH2**→**CLB2**	**GAS1**→**SWI4**
**SWI5**→**FKH1**	**DPB3**→**CDC45**	**DPB2**→**DPB3**	CLN2→CLN3
ASF1→HHF1	GAS1→KRE6	CLN3→CLB6	CDC14→SIC1
SWI4→MBP1	MSH6→POL30	CLB6→CLN1	SWI4→CHS3
KAR3→NUM1	HHF1→HHT1	MOB1→DBF2	RFA1→RFA3
CLB1→CLB3	CLN1→CLN3	CDC45→CDC6	CLB1→CLB5
HHF1→HTB2	HPR5→RAD54		
			
**BN Without prior knowledge**			
**HTA1**→**HHT1**	**FUS1**→**FAR1**	**FKH2**→**CLB2**	**GAS1**→**SWI4**
**SWI5**→**FKH1**	**DPB3**→**CDC45**	**DPB2**→**DPB3**	CLN3→CLN2
DBF4→CDC5	CDC8→CIK1	CDC6→CDC45	CLB3→CDC6
**SIC1**→**CDC14**			

Relationships in bold font are predicted both with and without prior knowledge.

**Table 5:** Predicted yeast gene regulatory relationships that are confirmed by ChIP-chip

**BN with prior knowledge**			
**FKH2**→**HHF1**	**FKH2**→**CLB2**	**SWI6**→**CLN1**	**FKH2**→**HHT1**
**SWI5**→**FKH1**	**SWI6**→**HO**	**SWI6**→**POL30**	**SWI4**→**MFA2**
FKH1→SWE1	FKH2→CDC6	FKH2→SWI4	SWI4→PSA1
SWI6→HHT1	SWI5→ASH1	SWI6→CLN2	FKH2→SWE1
FKH2→HPR5	SWI6→RAD54	FKH1→RAD51	SWI6→HHF1
SWI6→AGA1	SWI4→AGA1	SWI4→MBP1	
			
**BN without prior knowledge**			
**SWI6**→**POL30**	**SWI6**→**CLN1**	**FKH2**→**HHT1**	**SWI5**→**FKH1**
**FKH2**→**HHF1**	**SWI4**→**MFA2**	**SWI6**→**HO**	**FKH2**→**CLB2**
**SWI4**→**TIR1**	FKH1→CDC6	FKH1→CDC20	

Relationships in bold font are predicted both with and without prior knowledge.

Evidently, our method is capable of identifying a higher number of the positive benchmarks compared with the plain BN without prior knowledge. When evaluated with the BIND annotation, the number of correctly identified interactions doubled from 13 to 26 (p~0.13, χ^2^~2.28). The plain BN actually did not perform better than random selection (p~0.11). In contrast, BN with prior knowledge performed significantly better than random selection with χ^2 ^= 24.5, p < 0.001. When evaluated with the ChIP-chip data, the story is similar. The number of correctly identified gene regulatory relationships increased from 11 to 23 with the addition of prior knowledge (p < 0.01, χ^2 ^= 6.71). Without the prior knowledge, the plain BN is not different from random selection (p~0.1).

**Figure 5:**
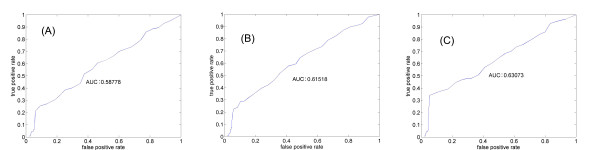
**ROC curves for the network modeling of the yeast cell cycle data using plain BN (A), Werhli and Husmeier's (B), and our algorithm (C)**. ChIP-chip binding data were used as benchmark. Adding prior knowledge significantly improved BN performance at identifying the TF-target pairs.

Figure 5A-5C shows the ROC curves that give a more quantitative view of the performance of BN with/without prior knowledge, and of the Werhli and Husmeier's algorithm [22,23], in detecting TF-target gene interactions. Incorporation of prior knowledge significantly improved the performance with higher AUC. Our algorithm performed slightly better than Werhli and Husmeier's.

### Validation using mouse pancreas development data

**Figure 6:**
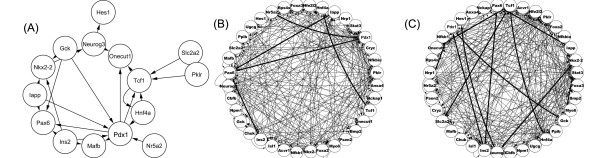
**The pancreas development network already established by existing experiments (A), predicted by the plain BN (B), and by BN + prior knowledge (C)**. The bold edges in (B) and (C) are those that overlap with the edges in (A).

**Figure 7:**
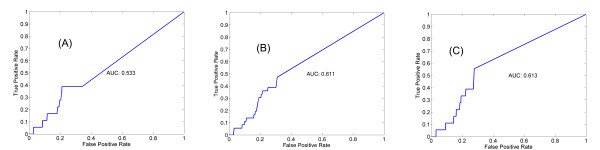
**ROC curves for the pancreas development data with plain BN (A), Werhli and Husmeier's algorithm (B), and our approach (C)**. 24 experimentally confirmed interactions were used as benchmark.

We also validated our algorithm using a mammal dataset. The experiment profiled gene expression changes in pancreas during embryonic development or during compensatory growth after partial pancreatectomy. Elucidating the networks is key to understand the complex nature of pancreas development and function [50,51]. A number of efforts have been made to manually annotate the key transcription factors and the gene networks they regulate based on low-throughput data, nicely reviewed by Servitja and Ferrer [52]. In Table 6, we list the 24 experimentally confirmed gene-gene regulatory relationships [52], and their network is depicted in Figure 6A. With prior knowledge BN modeling of the expression data is able to recover half of them (12), as shown in Figure 6C and Table 6. In contrast, the plain BN is only able to identify 6 of them (Figure 6B). This is again a ~two-fold enhancement. In Figures 7A-7C the ROC curves are presented. Incorporation of prior knowledge significantly improved the ability to detect known interactions. Our algorithm performed comparably to Werhli and Husmeier's.

**Table 6:** Established pancreas gene regulatory relationships that are identified by BN modeling

Known regulatory relationship	Identified by BN modeling with prior knowledge	Identified by the plain BN without prior knowledge
Hes1→Neurog3	√	
Hnf4a→Tcf1	√	√
Pdx1→Gck	√	
Pdx1→Hnf4a		
Pdx1→Iapp		
Pdx1→Ins2	√	
Pdx1→Nr5a2	√	√
Mafb→Ins2		
Mafb→Pdx1		
Neurog3→Nkx2-2	√	
Nkx2-2→Gck	√	√
Nkx2-2→Iapp		
Nkx2-2→Ins2		
Onecut1→Pdx1		
Onecut1→Neurog3		
Onecut1→Tcf1	√	
Pax6→Gck		
Pax6→Iapp	√	√
Pax6→Ins2	√	√
Pax6→Pdx1	√	√
Tcf1→Hnf4a		
Tcf1→Pdx1		
Tcf1→Pklr		
Tcf1→Slc2a2	√	

BN with prior knowledge can recover half of the experimentally confirmed transcriptional regulations during mouse pancreas development, two times more than the plain BN without prior knowledge.

In Additional file 1, we listed the GO similarity and PubMed co-citation of the gene pairs with known regulatory relationships that were missed by plain BN. Clearly, almost all of them have high GO similarity and share a significant number of co-citations. Adding the functional linkage as prior knowledge helped to recover them.

## Discussion

In this study we proposed a new algorithm to quantitatively utilize prior biological knowledge in the network modeling of gene expression data. First the functional linkage of gene pairs was assessed based on multiple data sources using the naïve Bayesian classifier. The result was then utilized to construct a candidate network edge reservoir, where the number of replicate edges between each gene pair was proportional to their function linkage probability. During simulation new candidate network structure was formed by sampling from this reservoir at each iteration. Since the edges of gene pairs with stronger functional linkage had more representations in the reservoir, these biologically meaningful edges enjoyed a preferential treatment in network simulation. With both the simulated and real gene expression data, we demonstrated that incorporating the prior knowledge significantly improved the network modeling performance. More information of the gene interaction network could be extracted from the microarray data with higher accuracy. In contrast, in all datasets, without the prior knowledge, though the number of benchmark regulations recovered is more than a random selection, the improvement is not statistically significant, demonstrating the necessity to supplement the gene expression data with additional information. This finding that plain BN did not perform better than random selection was not unexpected, similar observations was recently reported for a number of publically available reverse-engineering algorithms when gene expression data is the sole source of information [47].

Our algorithm provides a practical way to integrate the probabilistic biological knowledge that is different from previous efforts by others [2]. The quantitative nature makes it capable to handle soft constraints. Using the approach by Werhli and Husmeier for instance [22,23], we differ in several key steps. First, they encode multiple sources of prior knowledge in a weighted sum via an energy function; we integrate information from multiple sources through a Bayesian classifier. Furthermore, in our approach the MCMC samples from a candidate edge distribution defined by the prior knowledge, rather than from the network posterior distribution where the network prior is defined by the prior knowledge. Our algorithm utilizes the prior knowledge at interaction level, while theirs at the network level. Finally the Werhli and Husmeier approach is more computational intensive. To reduce the computational complexity, they sum over all parent configurations of each node and limit the number of parents of each node to 3 or less; the complexity of this operation is $\left(\begin{array}{c} N-1\\ m \end{array}\right)$ (where N is size of the network, and m the maximum FanIn) [23]. We find that it is still memory consuming for networks of moderate or large sizes. For instance, a Dell Optiplex 755 with 2GHZ DUO CPU, 3.25 GB RAM ran out of memory when simulating the 107-gene yeast network. Our algorithm does not have this problem.

We used two sources of prior evidence of functional linkage to assist network modeling: the PubMed co-citation and GO schematic similarity. However, our framework by design allows the integration of other types of data or knowledge, for instance, high throughput genomic data including PPI and ChIP-chip; gene-gene relationships derived from advanced methods including text mining [53], database curation, and computational modeling of sequence information; and many other sources. It has been demonstrated that the degree of improvement brought in by prior knowledge highly depends on the quality of the information being added [54]. Low quality prior knowledge could even lower the performance of BN [54]. Presently, most of the available prior knowledge each on its own suffers from high false positive rate and being incomplete, which can limit their efficacy in network modeling. Integration of data from different sources and utilizing their consensus provides an effective means to deal with this issue [1,2]. A caveat here is, when considering more sources of data, the inter-dependency among them need to be scrutinized more carefully, and maybe a more sophisticated integration method than the naïve Bayesian classifier is needed.

A number of different approaches have been developed to integrate multiple sources of prior information in the BN modeling of gene expression data, at the different steps of the simulation process [4,11-14]. It would be of interest to compare the efficiency of the different approaches, investigate whether the optimal approach depends on the types of prior knowledge, and if the different approaches can be combined for a most efficient utilization of prior knowledge in network modeling.

## Conclusion

In this paper we proposed a new algorithm to integrate and utilize the prior biological knowledge in the BN modeling of gene expression data. Our study demonstrated that incorporating prior knowledge at the step of network structure simulation is an efficient way to preserve the quantitative information in it, and to improve the performance of network modeling.

## Methods

### Preparation of gene expression data for algorithm validation

#### Simulated data

The simulated time course gene expression dataset was generated using SynTReN [46] for a artificial network with 76 genes, of which 24 act as regulators with a total of 124 regulatory relationships (*i.e*. 124 edges). The total number of time points is 50. All parameters of SynTReN were set to default values [46], except number of correlated inputs, which was set to 50%. The topological structure and inner interacting relationships are sampled from the characteristics of the yeast transcriptional network, therefore the results will be indicative of the algorithm performance on real data.

#### Yeast cell cycle study

Yeast cell cycle gene expression data were downloaded from http://genome-www.stanford.edu/cellcycle/. These studies [48,55] profiled expression changes in 6178 genes at ~20 time points under each condition following alpha factor arrest (18 time points from 0-119 minutes), elutriation ELU (14 time points from 0-390 minutes), and arrest of a cdc15 (24 time points from 10-290 minutes) and a cdc28 (28 time points from 0-160 minutes) temperature sensitive mutant. Many genes have missing data points. The cdc28 data is the most severely affected, ~80% of genes contains at least 1 missing values. For the remaining three datasets, it ranged 6-27%. In this study, we chose the cdc15 dataset, as it contains the most number of time points out of the three [56]. Network modeling was performed on the 107 known cell cycle genes [57]. The list is given in Table 7. These are the genes that most likely to have interesting interactions during the time course being studied.

**Table 7:** The 107 Yeast cell cycle genes that were simulated for their network structure

ACE2 (850822)	CLB6 (853003)	HHF2 (855701)	MSH6 (851671)	RFA3 (853266)
AGA1 (855780)	CLN1 (855239)	HHT1 (852295)	MST1 (853640)	RME1 (852935)
ASE1 (854223)	CLN2 (855819)	HHT1 (855700)	NDD1 (854554)	RNR1 (856801)
ASF1 (853327)	CLN3 (851191)	HHT2 (852295)	NUM1 (851727)	RNR3 (854744)
ASF2 (851330)	CTS1 (850992)	HHT2 (855700)	PCL1 (855427)	SED1 (851649)
ASH1 (853650)	CWP1 (853766)	HO (851371)	PCL2 (851430)	SIC1 (850768)
CDC14 (850585)	CWP2 (853765)	HSL1 (853760)	PCL9 (851375)	SPC42 (853824)
CDC20 (852762)	DBF2 (852984)	HTA1 (851811)	PDS1 (851691)	SPO12 (856557)
CDC21 (854241)	DBF4 (851623)	HTA2 (852283)	PMS1 (855642)	SST2 (851173)
CDC45 (850793)	DPB2 (856305)	HTB1 (851810)	POL1 (855621)	STE2 (850518)
CDC5 (855013)	DPB3 (852580)	HTB2 (852284)	POL12 (852245)	SWE1 (853252)
CDC6 (853244)	EGT2 (855389)	KAR3 (856263)	POL2 (855459)	SWI4 (856847)
CDC8 (853520)	FAR1 (853283)	KAR4 (850303)	POL30 (852385)	SWI5 (851724)
CDC9 (851391)	FKH1 (854675)	KIN3 (851273)	PRI1 (854825)	SWI6 (850879)
CHS1 (855529)	FKH2 (855656)	KRE6 (856287)	PRI2 (853821)	TEC1 (852377)
CHS3 (852311)	FKS1 (851055)	MBP1 (851503)	PSA1 (851504)	TIP1 (852359)
CIK1 (855238)	FUS1 (850330)	MCD1 (851561)	RAD17 (854550)	TIR1 (856729)
CLB1 (853002)	GAS1 (855355)	MCM1 (855060)	RAD27 (853747)	UNG1 (854987)
CLB2 (856236)	GIC2 (851904)	MFA2 (855577)	RAD51 (856831)	YRO2 (852343)
CLB3 (851400)	HHF1 (852294)	MNN1 (856718)	RAD54 (852713)	
CLB4 (850907)	HHF1 (855701)	MOB1 (854700)	RFA1 (851266)	
CLB5 (856237)	HHF2 (852294)	MSH2 (854063)	RFA2 (855404)	

In parenthesis are the corresponding gene IDs.

#### Mouse pancreas development and regeneration after damage

The pancreas development and growth expression data was downloaded from the RNA Abundance Database http://www.cbil.upenn.edu/RAD, with study IDs 2 and 1790. Study 2 profiled mouse pancreas gene expression at six different developmental time points: embryonic day 14.5, 16.5, 18.5, at birth, at postnatal day 7, and at adulthood. 4 samples at E14.5, and 6 at all the following time points, totaling 34 samples. Study 1790 profiled gene expression in mice pancreas following partial pancreatectomy and Exendin-4 treatment. Exendin-4 is a glucagon-like peptide-1 receptor agonist that augments the pancreatic islet beta-cell mass by increasing beta-cell neogenesis and proliferation and by reducing apoptosis. Mice underwent 50% pancreatectomy or sham operation, and received Exendin-4 or vehicle every 24 hours. 3-4 animals from each group were sacrificed at each time point of 12, 24 and 48 hr after operation, together with 4 animals that received no operation, totaling 46 samples. Because the two studies each only contain a few time points, we combined their data for network modeling [58]. Replicate samples under the same condition at the same time point were averaged.

The network modeling was performed on 36 genes manually collected from a recent review by Servitja and Ferrer [52], which are known to be important in pancreas development. They are listed in Table 8.

**Table 8:** The 36 mouse genes chosen to reconstruct interaction networks during pancreas development and growth

Acvr1 (11477)	Hes1 (15205)	Nfe2l2 (18024)	Pdx1 (18609)
Anxa4 (11746)	Hnf4a (15378)	Nfkb1 (18033)	Pklr (18770)
Bmp2 (12156)	Iapp (15874)	Nfkbia (18035)	Ppib (19035)
Cbfb (12400)	Ins2 (16334)	Nkx2-2 (18088)	Psen2 (19165)
Chuk (12675)	Isl1 (16392)	Npm1 (18148)	Rps4x (20102)
Cryz (12972)	Mafb (16658)	Nr5a2 (26424)	Slc2a2 (20526)
Foxa2 (15376)	Myo6 (17920)	Nrp1 (18186)	Stat3 (20848)
Foxa3 (15377)	Nckap1 (50884)	Onecut1 (15379)	Tcf1 (21405)
Gck (103988)	Neurog3 (11925)	Pax6 (18508)	Ugcg (22234)

In parenthesis are the corresponding gene IDs.

#### Digitization of gene expression data

Expression data were further discretized into three levels. In each data set, we calculated the mean (μ) and standard deviation (SD) of expression across all time points for each gene. Each expression value is then assigned to 0, 1 or 2 according to whether the value is less than μ-SD, between μ-SD and μ+SD, or above μ+SD.

#### Prior data of interaction and transcription binding

Annotations of known yeast gene interaction were downloaded from the Biomolecular Interaction Network Database (BIND, http://bind.ca), a database designed to store full descriptions of interactions, molecular complexes and pathways [49]. BIND includes both directed (such as protein-DNA interaction) and un-directed (such as protein-protein interaction) interactions. Therefore when comparing to BIND annotations, we ignored direction.

Simon *et al *studied the transcription regulation of yeast genes by 9 cell cycle regulating transcription factors (TF): Fkh1, Fkh2, Ndd1, Mcm1, Ace2, Swi5, Mbp1, Swi4, and Swi6, using the ChIP-chip technology [45]. These nine TFs are among the 107 cell cycle genes that we performed network modeling. The data were downloaded from http://staffa.wi.mit.edu/cgi-bin/young_public/navframe.cgi?s=17&f=downloaddata. For each TF, the study derived a binding p-value for each gene which reflects the likelihood that the TF binds to the promoter of this gene. We constructed a positive control target set for each TF that consists of those with *p *< 0.001, a negative control target set for each TF that consists of those with *p *> 0.1. Note that the transcription binding data provide directed information.

## List of abbreviations used

AUC: area under curve; BN: Bayesian Network; DAG: directed acyclic graph; GO: Gene Ontology; MCMC: Markov Chain Monte Carlo; PPI: protein-protein interaction; ROC: receiver operating characteristic; TF: transcription factor.

## Authors' contributions

SG, and XW designed the study. SG wrote the algorithms, performed the analysis, and created the figures and tables. SG and XW wrote the manuscript, read and approved the final version of the manuscript.

## Supplementary Material

Additional file 1 **Predicted regulatory relationships missed by the plain BN**. most established regulatory relationships missed by the plain BN involve two genes that share significant GO similarity and PubMed co-citation. Click here for file
