# Supplementary material for: Quantitative utilization of prior biological knowledge in the Bayesian network modeling of gene expression data
Source: BMC Bioinformatics. 2011 Aug 31;12:359. doi: 10.1186/1471-2105-12-359 (PMC3203352; doi:10.1186/1471-2105-12-359)
Supplement: Additional file 1 — Predicted regulatory relationships missed by the plain BN. most established regulatory relationships missed by the plain BN involve two genes that share significant GO similarity and PubMed co-citation. [file 1471-2105-12-359-S1.DOCX]

| **regulator** | **target** | **GO similarity** | **-log_10_(p_PubMed_)** |
| --- | --- | --- | --- |
| **Yeast cell cycle, BIND** | | | |
| ASF1 | HHF1 | 0.7 | 5.67 |
| CDC14 | SIC1 | 1 | 2.95 |
| CDC45 | CDC6 | 1 | 11.76 |
| CLB1 | CLB3 | 1 | >30.00 |
| CLB1 | CLB5 | 1 | 3.37 |
| CLB6 | CLN1 | 1 | 3.88 |
| CLN1 | CLN3 | 1 | 3.19 |
| CLN2 | CLN3 | 1 | 12.63 |
| CLN3 | CLB6 | 1 | 3.01 |
| GAS1 | KRE6 | 1 | 3.60 |
| HHF1 | HHT1 | 1 | >30.00 |
| HHF1 | HTB2 | 1 | >30.00 |
| HPR5 | RAD54 | 1 | 3.03 |
| KAR3 | NUM1 | 0.082 | 3.24 |
| MOB1 | DBF2 | 1 | >30.00 |
| MSH6 | POL30 | 1 | 4.99 |
| RFA1 | RFA3 | 1 | >30.00 |
| SWI4 | CHS3 | 0 | 5.94 |
| SWI4 | MBP1 | 1 | >30.00 |
|  | | | |
| **Yeast cell cycle, ChIP-chip** | | | |
| FKH1 | SWE1 | 1 | 3.30 |
| FKH2 | CDC6 | 0.074 | 3.46 |
| FKH2 | HPR5 | 1 | 3.33 |
| FKH2 | SWE1 | 1 | 3.23 |
| FKH2 | SWI4 | 1 | 3.18 |
| SWI4 | AGA1 | 0 | 3.33 |
| SWI4 | MBP1 | 1 | >30.00 |
| SWI4 | PSA1 | 0 | 6.63 |
| SWI5 | ASH1 | 1 | 3.19 |
| SWI6 | CLN2 | 1 | 6.50 |
| SWI6 | HHF1 | 0.051 | 3.30 |
| SWI6 | HHT1 | 1 | 3.24 |
| SWI6 | RAD54 | 1 | 3.00 |
|  | | | |
| **Pancreas development** | | | |
| Pdx1 | Ins2 | 1 | >30.00 |
| Neurog3 | Nkx2-2 | 1 | 13.06 |
| Tcf1 | Slc2a2 | 0.05 | 12.70 |
| Hes1 | Neurog3 | 1 | 9.73 |
| Pdx1 | Gck | 0.38 | 8.14 |
| Onecut1 | Tcf1 | 1 | >30.00 |
